# *Burkholderia pseudomallei* Evades Nramp1 (Slc11a1)- and NADPH Oxidase-Mediated Killing in Macrophages and Exhibits Nramp1-Dependent Virulence Gene Expression

**DOI:** 10.3389/fcimb.2017.00350

**Published:** 2017-08-08

**Authors:** Veerachat Muangsombut, Patoo Withatanung, Varintip Srinon, Narisara Chantratita, Mark P. Stevens, Jenefer M. Blackwell, Sunee Korbsrisate

**Affiliations:** ^1^Department of Immunology, Faculty of Medicine Siriraj Hospital, Mahidol University Bangkok, Thailand; ^2^Microbiology Laboratory, Veterinary Diagnostic Center, Faculty of Veterinary Science, Mahidol University Nakhon Pathom, Thailand; ^3^Department of Microbiology and Immunology, Faculty of Tropical Medicine, Mahidol University Bangkok, Thailand; ^4^Division of Infection and Immunity, The Roslin Institute and Royal (Dick) School of Veterinary Studies, University of Edinburgh Midlothian, United Kingdom; ^5^Telethon Kids Institute, The University of Western Australia Subiaco, WA, Australia; ^6^Department of Pathology, University of Cambridge Cambridge, United Kingdom

**Keywords:** *B. pseudomallei*, *B. thailandensis*, iron, macrophages, Nramp1, Slc11a1, secretion system

## Abstract

Bacterial survival in macrophages can be affected by the natural resistance-associated macrophage protein 1 (Nramp1; also known as solute carrier family 11 member a1 or Slc11a1) which localizes to phagosome membranes and transports divalent cations, including iron. Little is known about the role of Nramp1 in *Burkholderia* infection, in particular whether this differs for pathogenic species like *Burkholderia pseudomallei* causing melioidosis or non-pathogenic species like *Burkholderia thailandensis*. Here we show that transfected macrophages stably expressing wild-type Nramp1 (Nramp1^+^) control the net replication of *B. thailandensis*, but not *B. pseudomallei*. Control of *B. thailandensis* was associated with increased cytokine responses, and could be abrogated by blocking NADPH oxidase-mediated production of reactive oxygen species but not by blocking generation of reactive nitrogen species. The inability of Nramp1^+^ macrophages to control *B. pseudomallei* was associated with rapid escape of bacteria from phagosomes, as indicated by decreased co-localization with LAMP1 compared to *B. thailandensis*. A *B. pseudomallei bipB* mutant impaired in escape from phagosomes was controlled to a greater extent than the parent strain in Nramp1^+^ macrophages, but was also attenuated in Nramp1^−^ cells. Consistent with reduced escape from phagosomes, *B. thailandensis* formed fewer multinucleated giant cells in Nramp1^+^ macrophages at later time points compared to *B. pseudomallei. B. pseudomallei* exhibited elevated transcription of virulence-associated genes of Type VI Secretion System cluster 1 (T6SS-1), the Bsa Type III Secretion System (T3SS-3) and the *bimA* gene required for actin-based motility in Nramp1^+^ macrophages. Nramp1^+^ macrophages were found to contain decreased iron levels that may impact on expression of such genes. Our data show that *B. pseudomallei* is able to evade Nramp1- and NADPH oxidase-mediated killing in macrophages and that expression of virulence-associated genes by pathogenic *B pseudomallei* is enhanced in macrophages expressing wild-type compared to non-functional Nramp1. *B. thailandensis* has been proposed as surrogate for *B. pseudomallei* in the study of melioidosis however our study highlights important differences in the interaction of these bacteria with macrophages.

## Introduction

The Natural resistance-associated macrophage protein 1 (Nramp1), also known as solute carrier family 11 member a1 (Slc11a1), acts as a protein-coupled metal transporter for delivering divalent cations (i.e., Fe^2+^, Zn^2+^, and Mn^2+^) across the membrane of late endosomes/lysosomes (Blackwell et al., 2000). One explanation for the ability of Nramp1 to control intracellular bacterial infections is by limiting availability of divalent cations within phagosomes and thus restricting bacterial growth (Cherayil, 2011). For example, in *Mycobacterium bovis-*infected macrophages, Nramp1 and the iron exporter ferroportin 1 (Fpn1; Slc40a1) are recruited to *Mycobacterium*-containing phagosomes, leading to transportation of iron outside macrophages and suppression of *M. bovis* growth (Van Zandt et al., 2008). In addition, Nramp1 can promote host immune responses *via* regulation of iron homeostasis. Nramp1-mediated reduction of cellular iron pools results in suppression of interleukin-10 (IL-10), an anti-inflammatory cytokine, leading to high TNF-α production and enhanced killing activity of *S*. Typhimurium-infected macrophage *via* iNOS-mediated NO release (Fritsche et al., 2008). Despite numerous studies on bacterial survival in macrophages, the influence of Nramp1 on expression of bacterial virulence factors, either directly or through its pleiotropic effects on macrophage function, has not been addressed.

*Burkholderia pseudomallei* is a Gram-negative intracellular bacterium that causes melioidosis, a severe invasive disease of humans that is endemic in subtropical areas. It is an environmental saprophyte and can survive in water and soil for long periods. It is closely related to *Burkholderia thailandensis*, which has been found in a similar habitat to *B. pseudomallei*, but is rarely reported to cause disease in humans, though at high doses in a murine model it could cause pneumonia-derived sepsis (Glass et al., 2006). *B. pseudomallei* is able to survive and replicate in macrophages, and is able to invade non-phagocytic cells, spread from cell-to-cell *via* actin-based motility and induce cell fusion. *B. thailandensis* is less efficient than *B. pseudomallei* in its ability to adhere to and invade human lung epithelial cells, resulting in lower host cell damage (Kespichayawattana et al., 2004). Although a number of studies reported the roles of Nramp1 expression in macrophages against bacterial infection (Nairz et al., 2014; Wessling-Resnick, 2015), the influence of Nramp1 in response to infection by *Burkholderia* species has not been reported.

Here we examine whether different Nramp1-controlled macrophage defense mechanisms act differently on *B. thailandensis* compared to *B. pseudomallei*. We examine the survival of the two *Burkholderia* spp. in stably transfected macrophage cell lines expressing wild-type (Nramp1^+^) and mutant (Nramp1^−^) Nramp1. We provide evidence that Nramp1 mediates killing of *B. thailandensis via* the production of reactive oxygen species. In contrast, intracellular net replication of *B. pseudomallei* was higher than observed for *B. thailandensis* in Nramp1^+^ macrophages. Our data indicate that evasion of Nramp1- and NADPH oxidase-mediated killing of *B. pseudomallei* in macrophages is associated with escape from phagosomes. Interestingly, even though *B. pseudomallei* escaped phagosomes to a comparable extent in macrophages with or without functional Nramp1, transcription of virulence genes associated with Type III and Type VI secretion systems and actin-based motility was elevated in Nramp1^+^ cells.

## Materials and methods

### Bacterial strains, macrophages cell lines, and growth conditions

Non-pathogenic *B. thailandensis* E264 and pathogenic *B. pseudomallei* K96243 strains were used throughout the study. A *B. pseudomallei* mutant lacking the Bsa T3SS translocon component BipB was constructed by insertion of the pKNOCK-Tet suicide vector and has been described (Suparak et al., 2005). *Salmonella enterica* serovar Typhimurium 13311 (hereinafter referred to as *S*. Typhimurium) was obtained from the American Type Culture Collection (ATCC). Bacteria were routinely cultured on Luria–Bertani (LB; TM MEDIA, India) agar or in broth. The *bipB* mutant was maintained on agar plates or in broth containing 60 μg/mL tetracycline (Sigma-Aldrich, USA). Growth of *Burkholderia* strains under iron-limiting conditions was performed by culturing bacteria in M9 minimal salts supplemented with 0.1% (w/v) casamino acids and 0.4% glucose (M9CG) as previously described (Burtnick and Brett, 2013). All experiments involving viable *B. pseudomallei* were conducted under biosafety level 3 containment.

Stably transfected RAW 264.7 macrophage cell lines expressing wild-type (Nramp1^+^) and G169D mutant (Nramp1^−^) Nramp1 were constructed as previously described (White et al., 2004), and obtained from the laboratory of Prof. Jenefer M. Blackwell. In transfected macrophages expressing wild-type Nramp1 the protein localizes to membranes of late endosomes/lysosomes, whereas the G169D mutant protein is retained in the endoplasmic reticulum and degraded (White et al., 2004). RAW 264.7 macrophages carry a mutated endogenous copy of the Nramp1 gene (White et al., 2004). The cell lines were routinely maintained in RPMI 1640 medium (Gibco, USA) supplemented with 10% (v/v) heat-inactivated fetal bovine serum (FBS; HyClone, USA) at 37°C under an atmosphere of 5% CO_2_, and periodically grown under 400 μg/mL geneticin (G418 sulfate; Sigma-Aldrich, USA) selection to ensure that macrophage clones were stably expressing the Nramp1 transgene.

### Macrophage activation and inhibitor treatments

Sixteen hours prior to infection experiments, macrophages were routinely activated using 50 U/mL of recombinant mouse IFN-γ (Sigma-Aldrich, USA). A dose-response experiment for IFN-γ treated cells was also performed for bacterial survival assays. Activated macrophages (50 U/mL IFN-γ) were incubated with the NADPH oxidase inhibitor diphenyleneiodonium chloride (DPI; Sigma-Aldrich, USA) at 50 μg/mL, or the specific iNOS inhibitor aminoguanidine (AG; Sigma-Aldrich, USA) at 250 μg/mL, at 37°C, 5% CO_2_ for 1 h prior to bacterial infection. At these inhibitor concentrations, cell viability was more than 95% as determined by MTT (3-(4,5-dimethylthiazol-2-yl)-2,5-diphenyltetrazolium bromide; Sigma-Aldrich, USA assay.

### Macrophage infection assay

Net intracellular survival and replication of bacteria in macrophages was quantified by means of net survival where bacterial numbers decline over time or net replication where numbers increase over time to reflect the balance of intracellular killing or intracellular replication processes as previously described (Muangsombut et al., 2008). Briefly, IFN-γ stimulated (50 U/mL, or dose response as indicated) Nramp1^+^ or Nramp1^−^ macrophages were seeded at a density of 4 × 10^5^ cells into 24-well tissue culture plates and cultured for 16 h. Activated macrophages were infected at a multiplicity of infection (MOI) of 1 and incubated at 37°C, 5% CO_2_. After 1 h of infection, extracellular *Burkholderia* spp. or *S*. Typhimurium were killed by incubation with culture medium containing 250 μg/mL kanamycin (for *Burkholderia* spp.) or 100 μg/mL gentamicin (for *S*. Typhimurium) for 2 h at 37°C under 5% CO_2_ atmosphere. At this point, in some macrophages infection experiments, cells were lysed to enumerate bacteria that had been taken up by the macrophages. For studies to quantify net replication over time after this point, the infected cells were maintained in culture medium containing 20 μg/mL kanamycin or 10 μg/mL gentamicin. At the indicated time points, the intracellular bacteria were released by adding 0.1% Triton X-100 (Sigma-Aldrich, USA). The number of intracellular bacteria recovered were quantified by serial dilution and plating on tryptic soy agar (TSA). Bacterial colonies were counted after 24–48 h of incubation at 37°C.

### Intracellular oxidative burst and nitric oxide detection assays

Intracellular superoxide anion levels were determined by measuring deposition of formazan inside macrophages following addition of nitroblue tetrazolium (NBT) (Rook et al., 1985). Briefly, IFN-γ (50 U/mL) activated infected cells were treated with freshly prepared 2 mg/mL NBT (Sigma-Aldrich, USA) and incubated at 37°C for 1 h. As a positive control, macrophages were triggered with 0.5 μg/mL of phorbol myristate acetate (PMA; Sigma-Aldrich, USA) for 1 h. To terminate the reaction, the supernatant was removed and the cells were washed twice with methanol. The reduced NBT formazan deposits inside the cells were solubilized by addition of 120 μL of 2 M potassium hydroxide (KOH; Merck) and 140 μL of dimethyl sulfoxide (DMSO; Sigma-Aldrich, USA). Absorbance of the solution was determined at 630 nm. Background values were determined by incubation of macrophages in medium including NBT without PMA. Cell numbers were determined colorimetrically using 0.5% crystal violet in 50% ethyl alcohol and measuring absorbance at 590 nm. Oxidative burst-generated superoxide anion was represented as a ratio of formazan/crystal violet (A_630_/A_590_).

The generation of reactive nitrogen species (RNS) was measured as nitrite levels in cell culture supernatants, which represent the stable oxidation end-product of inducible nitric oxide synthase (iNOS)-generated nitric oxide (NO) (Breitbach et al., 2011). Briefly, IFN-γ (50 U/mL) stimulated macrophages were infected with bacteria as above. At 16 h post-infection (p.i.) nitrite levels were measured by addition of the Griess reagent (Molecular Probes, USA) and absorbance was read at 570 nm. The concentration of nitrite was determined from an established standard curve of sodium nitrite from 0 to 200 μM.

### Detection of bacterial virulence gene expression in infected macrophages

Transcription of the gene encoding the *Burkholderia* T6SS-1 cluster encoded effector Hcp1 (BPSS1498 for *B. pseudomallei* and BTH_II0868 for *B. thailandensis*), the gene encoding *Burkholderia* intracellular motility A (*bimA*_*Bps*_ BPSS1492; *bimA*_*Bth*_ BTH_II0875), and the gene encoding the Bsa T3SS-3 effector protein BopE and BsaU structural component (*bopE*_*Bps*_ BPSS1525; *bsaU*_*Bps*_ BPSS1539) in macrophages activated with 50 U/mL IFN-γ was determined by reverse transcription (RT)-PCR analysis. In brief, intracellular *Burkholderia* were released from the infected macrophages by isotonic lysis using deionized water and differential centrifugation. The pelleted bacteria were collected and total bacterial RNA was extracted using Presto^TM^ Mini RNA Bacterial kit (Geneaid, Taiwan) according to manufacturer's instruction. The conversion of total RNA to cDNA was performed using SuperScript® III First-Strand Synthesis System (Invitrogen, USA). The cDNA was subjected to DNA amplification with specific primers (Table 1). PCR reactions were performed under the following conditions: 97°C, 5 min and 30 cycles of 97°C for 45 s, 55°C for 45 s, and 72°C for 1 min, followed by incubation at 72°C for 5 min. Each assay was performed in duplicate. Amplification of *B. pseudomallei* and *B. thailandensis* 23S rRNA were used as an internal normalization control. The image densities of amplicons analyzed by gel electrophoresis were determined and normalized using ImageJ software.

**Table 1:** Oligonucleotide primers used in this study.

**Gene**	**Forward primer (5′ → 3′)**	**Reward primer (5′ → 3′)**	**Size (bp)**	**Reference**
*hcp1_*Bth*_*	CGCTGACCAAGGAGATCGAC	CGATACGTCTTCCATGTGGC	230	This study
*hcp1_*Bps*_*	GACGGATACAACAGGTGGGG	CCATTCGTCCAGTTTGCGG	228	This study
*bimA_*Bth*_*	AGGCGGGTAATCGACTCA	TTCGTCGTCCGACCATGA	87	Sitthidet et al., 2010
*bimA_*Bps*_*	GCGTTCAGCGAGCAA	CGATGCGCTGGTTCGT	280	This study
*bopE_*Bps*_*	CGGCAAGTCTACGAAGCGA	GCGGCGGTATGTGGCTTCG	393	Pumirat et al., 2010
*bsaU_*Bps*_*	AGGTGCGCT ACAGCTTCAAT	CGAATTCGACCG TCCATC	168	Pumirat et al., 2010
23S rRNA_Bth_	GTAGACCCGAAACCAGGTGA	CACCCCTATCCACAGCTCAT	115	Ngamdee et al., 2015
23S rRNA_Bps_	TTTCCCGCTTAGATGCTTT	AAAGGTACTCTGGGGATAA	320	This study

### Quantification of the intracellular iron content in infected macrophages

Total intracellular iron content of IFN-γ activated *Burkholderia*-infected macrophages was measured by Graphite Furnace Atomic Absorption Spectrometry (GFAAS), as previously described (Nairz et al., 2008), using AA-6800G (GFA-EX-7), atomic-absorption spectrometer with extended lifetime graphite tubes (Shimadzu corporation, Japan). *Burkholderia-*infected cells were washed three times in normal saline. The lysis of cells was carried out in 0.1% actual analytical nitric acid (Mallinckrodt Chemical, Ireland) and 0.2% Triton-X100 and ultrasonication for 40 s. A pyrolysis temperature of 1000°C and an atomization temperature of 2000°C were used. The concentration of total cellular protein was determined using Pierce™ bicinchoninic acid (BCA) protein assay kit (Thermo Scientific, USA) from an established standard curve of protein ranged from 0 to 2,000 μg/mL.

### Confocal analysis of bacterial co-localization with LAMP1

To assess the extent of *Burkholderia* escape from phagosomes of activated (50 U/mL IFN-γ) Nramp1^+^ or Nramp1^−^ macrophages, we examined co-localization of bacteria with lysosome-associated membrane protein 1 (LAMP1) at 4 h p.i. The bacteria were stained red with mouse anti-*Burkholderia* monoclonal antibody (9D5; 1:20; from the laboratory of Dr. Narisara Chantratita) and Alexa Fluor 568-goat anti-mouse IgG (1:500; Invitrogen, USA). LAMP1 was stained green with rat monoclonal antibody (1D4B; 1:200; Abcam, USA) and Alexa Fluor 488 goat anti-rat IgG antibody (1:500; Molecular Probes, USA), and nuclei were stained blue with 4,6-diamidino-2-phenylindole (DAPI; 1:500). All of the staining procedures were performed for 1 h at 37°C. Cells were examined by a laser-scanning confocal microscope equipped with LSM5 Image Browser (LSM 510 META, Carl Zeiss, Germany). The percentage of intracellular *Burkholderia* associated with LAMP1 was determined as the number of bacteria co-localized with LAMP1/total number of bacteria within macrophages × 100. The association of *Burkholderia* with LAMP1 was considered when the red fluorescent bacteria co-localized with the green fluorescence of LAMP1-positive vacuoles, represented as an area of yellow staining.

### Multinucleated giant cell (MNGC) formation assay

*Burkholderia*-infected macrophages were examined for MNGC formation according to a previously described method (Kespichayawattana et al., 2000). In brief, IFN-γ activated macrophages were infected as described above and at 4, 6, 8, and 10 h p.i., fixed and stained with Giemsa (Merck, Germany). By light microscopy at least 1,000 nuclei per slide were examined for MNGC formation. MNGC was defined as an infected cell with three or more nuclei per cell (Kespichayawattana et al., 2000). The percentage of MNGC formation was calculated as follows: (number of nuclei within multinucleated cells/total number of nuclei counted) × 100 (Kespichayawattana et al., 2000).

### Cytokine detection assay

Supernatants harvested from bacteria-infected macrophages were collected at indicated time point and filtered through a 0.45-μm membrane. Pro-inflammatory cytokines, including tumor necrosis factor alpha (TNF-α), interleukin-6 (IL-6) and IL-1β, were determined in triplicate by Luminex Bio-Plex^TM^ (Bio-Rad, USA) according to the instructions of the manufacturer. As a positive control for induction of pro-inflammatory cytokines, 20 μg/mL lipopolysaccharide (LPS; List Biological Labs, USA) from *Escherichia coli* was used.

### Statistical analysis

All assays were conducted at least in triplicate and unpaired *t*-tests of independent experiments were performed using Graph Pad Prism6 software. Results were considered significant at a two-tailed *P-*value of ≤ 0.05.

## Results

### Nramp1 controls replication of non-pathogenic *B. thailandensis*, but not pathogenic *B. pseudomallei*, within stably transfected RAW264.7 macrophages

**Figure 1:**
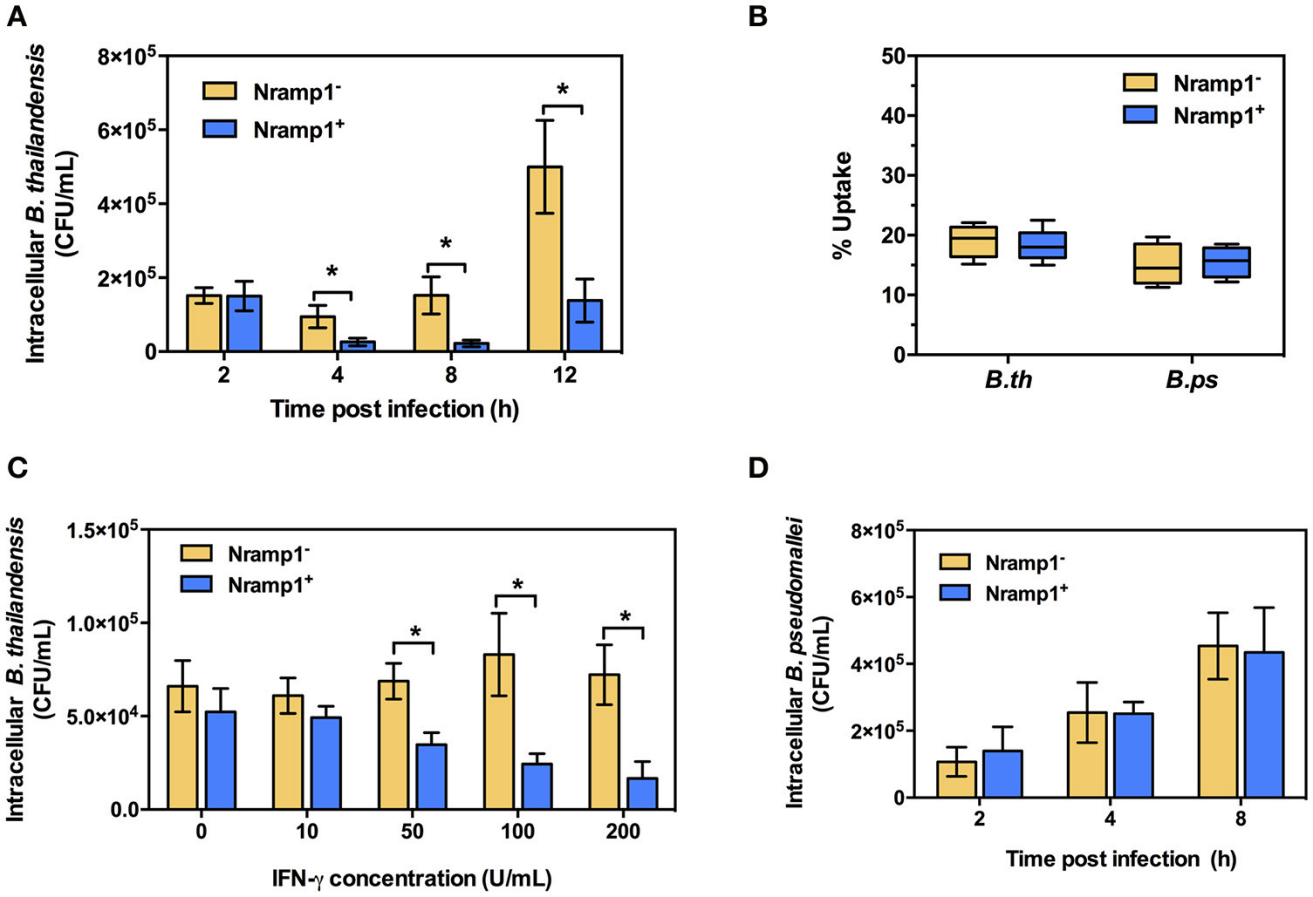
Effect of Nramp1 on the net intracellular survival of *Burkholderia* within macrophages. RAW264.7 macrophages expressing wild-type (Nramp1^+^) or mutated (Nramp1^−^) Nramp1 were infected with *Burkholderia* bacteria at a MOI of 1 for 1 h. Net intracellular survival and replication of **(A)** *B. thailandensis* or **(D)** *B. pseudomallei* in activated (50 U/mL IFN-γ) macrophages determined as bacterial colony-forming units (CFU) at the time points post-infection, as indicated. **(B)** shows percent uptake of the two bacterial species in activated (50 U/mL IFN-γ) Nramp1^+^ and Nramp1^−^ macrophages. **(C)** shows a dose-response analysis of IFNγ treatment on the efficiency of Nramp1-mediated macrophage antimicrobial activity against *B. thailandensis*, as determined by enumeration of viable antibiotic-protected intracellular bacteria at 6 h p.i. Results represent the mean ± standard error of the mean (SEM) of three independent experiments. Asterisk indicates statistical significance (*P* ≤ 0.05, *t*-test) when comparing Nramp1^+^ cells with Nramp1^−^ cells under the same conditions.

Since little was known about the effect of Nramp1 on *Burkholderia* spp. infection, we first compared the survival of *B. pseudomallei* with its closely related non-pathogenic bacteria *B. thailandensis* in activated (50 U/mL IFN-γ) RAW264.7 macrophages stably transfected with wild-type (Nramp1^+^) or mutant (Nramp1^−^) Nramp1 (White et al., 2004). Net intracellular survival of the bacteria was determined at 4, 8, and 12 h post-infection (p.i.). We observed that *B. thailandensis*-infected Nramp1^+^ cells exhibited a significantly reduced number of viable intracellular bacteria as compared with infected Nramp1^−^ cells at 4, 8, and 12 h p.i. (Figure 1A), indicating that Nramp1^+^ wild-type macrophages were more effective at killing *B. thailandensis* than Nramp1^−^ mutant macrophages.

Measurement of bacterial uptake showed no significant difference between Nramp1^+^ and Nramp1^−^ cells (Figure 1B), suggesting that the observed difference in killing was not due to differences in bacterial uptake between the macrophage lines. Moreover, we found that killing efficiency of Nramp1^+^ cells against *B. thailandensis* at 6 h p.i. was correlated with the concentration of IFN-γ used to stimulate the macrophages (from 10 to 200 U/mL) in a dose-dependent manner (Figure 1C), indicating that the influence of Nramp1 on controlling *B. thailandensis* survival is associated with IFN-γ stimulation. In contrast to these results with *B. thailandensis*, no significant difference in *B. pseudomallei* net intracellular replication was observed when comparing activated (50 U/mL IFN-γ) Nramp1^−^ and Nramp1^+^ macrophages (Figure 1D). The numbers of intracellular *B. pseudomallei* increased over the time intervals investigated in both types of macrophages suggesting that *B. pseudomallei* can escape the killing activity of functional Nramp1 in stably transfected RAW 264.7 macrophages.

### Nramp1 restricts *B. thailandensis* growth *via* an NADPH oxidase-mediated mechanism

**Figure 2:**
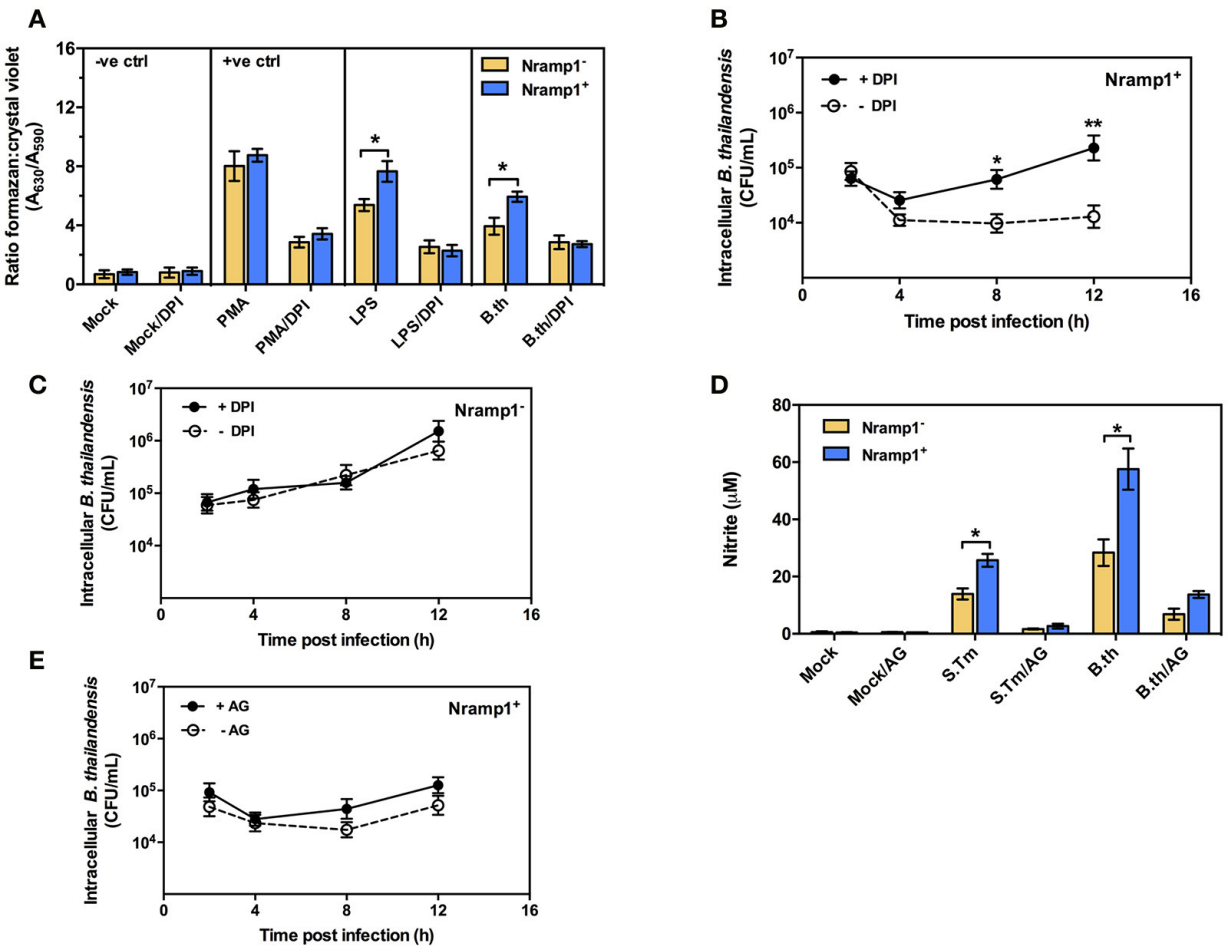
Effect of Nramp1 on ROS-mediated oxidative burst, RNS-mediated NO and killing of *B. thailandensis* within infected macrophages. **(A)** ROS-mediated oxidative burst in activated (50 U/mL IFN-γ) *B. thailandensis*-infected macrophages. Oxidative burst-generated superoxide anion production was determined by NBT reduction assay at 4 p.i. and represented as a ratio of formazan:crystal violet. Nramp1^+^ or Nramp1^−^ activated (50 U/mL IFN-γ) macrophages were treated with/without NADPH oxidase inhibitor (50 μg/mL of DPI) for 1 h prior to *B. thailandensis* infection. **(B,C)** show net intracellular survival of *B. thailandensis* in Nramp1^+^ cells or Nramp1^−^ cells, respectively, with/without inhibition (DPI) of the oxidative burst. **(D)** RNS-mediated NO of *B. thailandensis*-infected macrophages. The production of NO in infected macrophages was examined by Griess reagent assay. Nramp1^+^ or Nramp1^−^ cells were treated with/without iNOS enzyme inhibitor (250 μg/mL of AG) for 1 h prior to infection. **(E)** Net intracellular survival of *B. thailandensis* in Nramp1^+^ cells with/without AG inhibition of the iNOS pathway. Results represent the mean ± SEM of three independent experiments. Asterisk indicates statistical significance (*P* ≤ 0.05, *t*-test) when comparing Nramp1^+^ cells with Nramp1^−^ cells under the same conditions or when comparing infected cells treated with and without inhibitors.

Previous studies have shown that activation of Nramp1^+^ macrophages is associated with enhanced activation of the oxidative burst through ROS-mediated NF-κB activity and Lcn2 transcription (Fritsche et al., 2012). To determine whether infection with *B. thailandensis* is accompanied by enhanced oxidative burst activity in Nramp1^+^ macrophages, we measured the generation of superoxide anion. As shown in Figure 2A, *B. thailandensis*-infected or lipopolysaccharide (LPS)-stimulated Nramp1^+^ cells produce higher levels of superoxide anion than Nramp1^−^ cells at 4 h p.i., indicating a more effective induction of the oxidative burst by Nramp1^+^ cells. Superoxide anion is the precursor of more potent microbicidal ROS regulated by the NADPH oxidase complex. To determine whether production of ROS is important for Nramp1-mediated killing of *B. thailandensis*, we performed further experiments in the presence of the NADPH oxidase blocking agent diphenyleneiodonium (DPI). This resulted in a significant decrease in superoxide anion production in *B. thailandensis-*infected Nramp1^+^ cells (Figure 2A), concomitant with a failure in control of *B. thailandensis* replication (Figure 2B). This suggests that growth restriction of *B. thailandensis* within Nramp1^+^ cells occurs *via* the production of oxygen radicals by NADPH oxidase. DPI-treatment of Nramp1^−^ macrophages did not alter the growth of *B. thailandensis* (Figure 2C).

### Nramp1-mediated restriction of *B. thailandensis* does not involve nitric oxide (NO)

Previous studies have also indicated differences in induction of iNOS and associated NO production in activated Nramp1^+^ and Nramp1^−^ macrophages (Fritsche et al., 2008; Song et al., 2013). To determine the role of iNOS-induced RNS killing of *B. thailandensis* in Nramp1^+^ macrophages, we first measured the end product nitrite. We found that activated (50 U/mL IFN-γ) Nramp1^+^ cells infected with *B. thailandensis* or *S*. Typhimurium produced a significantly higher level of nitrite than activated Nramp1^−^ cells over 16 h p.i. (Figure 2D). However, while NO production was reduced by specific blocking of iNOS enzyme activity with aminoguanidine (AG) (Figure 2D), this had no statistically significant effect on the ability of Nramp1^+^ cells to control *B. thailandensis* (Figure 2E). These results indicate that the ability of Nramp1^+^ macrophages to kill *B. thailandensis* does not require iNOS-generated NO.

### *B. thailandensis* induces pro-inflammatory cytokine responses in Nramp1^+^ macrophages

**Figure 3:**
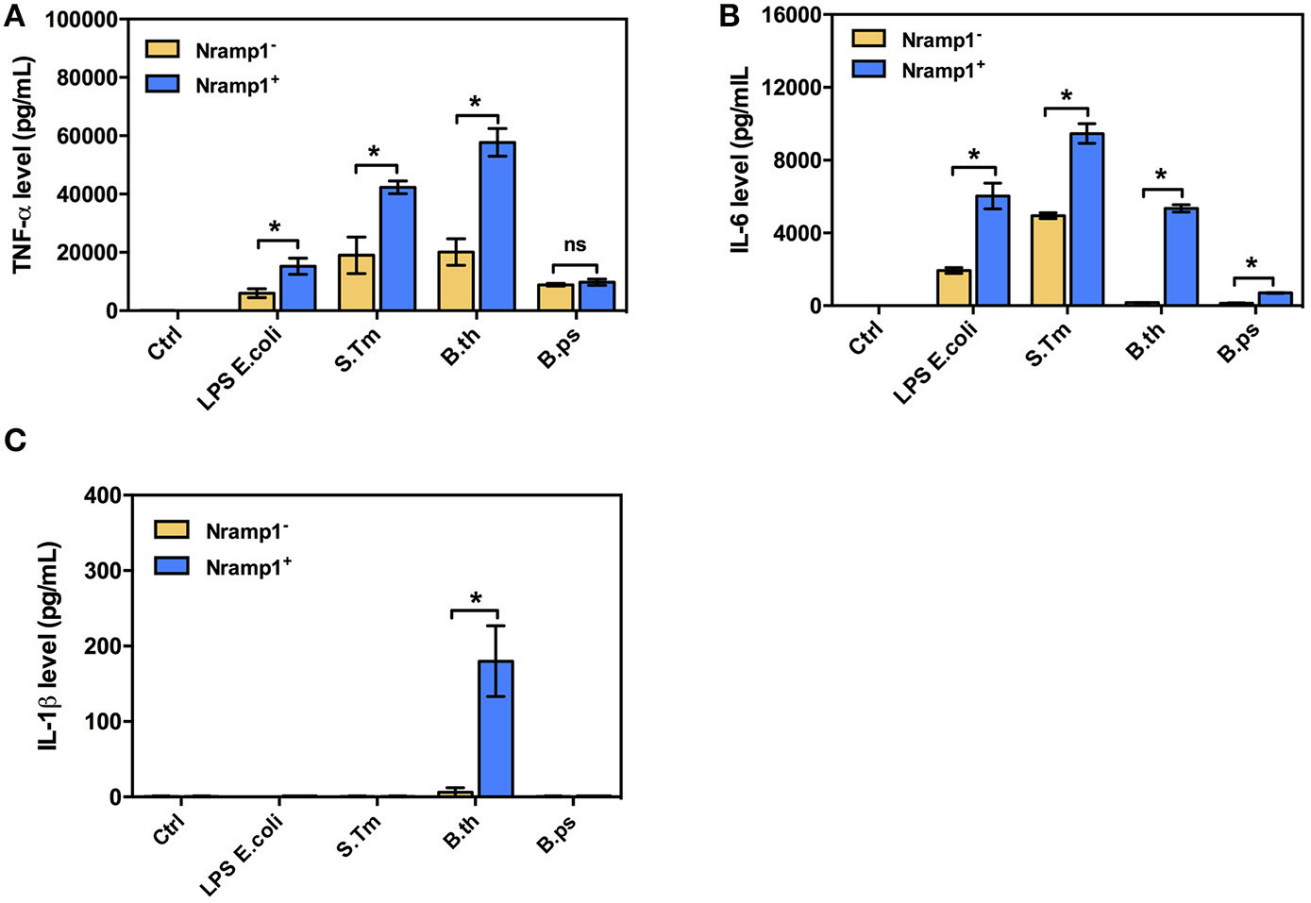
Cytokine profiles of macrophages expressing wild-type or mutated Nramp1 in response to *Burkholderia* infection. The secretion of proinflammatory cytokines by activated (50 U/mL IFN-γ) *Burkholderia*-infected macrophages: **(A)** tumor necrosis factor alpha (TNF-α), **(B)** interleukin-6 (IL-6) and **(C)** IL-1β. Supernatants were collected at 8 h post-stimulation, sterilized by filtration and analyzed for cytokine release by ELISA using Luminex Bio-Plex^TM^ (Bio-Rad). Results represent the mean ± SEM of three independent experiments. Asterisk indicates statistical significance (*P* ≤ 0.05, *t*-test) when comparing Nramp1^+^ cells with Nramp1^−^ cells under the same stimuli.

Since Nramp1 is well-known to have pleiotropic effects on release of pro-inflammatory cytokines, including IL1-β, TNF-α and IL-6, in response to bacterial uptake (Valdez et al., 2008), we also investigated the effects of Nramp1 on production of pro-inflammatory cytokines in response to *Burkholderia* infection. Activated (50 U/mL IFN-γ) *B. thailandensis*-infected Nramp1^+^ cells exhibited an increased level of TNF-α and IL-6 than Nramp1^−^ cells at 8 h post-stimulation (Figure 3). Expression of wild-type Nramp1 thus promotes an inflammatory cytokine response following infection with *B. thailandensis* (as well as in response to *S*. Typhimurium infection and LPS stimulation as controls) which was not observed following infection with *B. pseudomallei* (Figure 3) suggesting that this pathogen has a strategy to avoid the influence of functional Nramp1. Note also that *B. thailandensis*, but not *B pseudomallei*, was able to trigger an IL-1β response at 8 h p.i. in Nramp1^+^ but not Nramp1^−^ macrophages.

### *B. pseudomallei*, but not *B. thailandensis*, efficiently escapes from the phagosome of Nramp1^+^ macrophages

**Figure 4:**
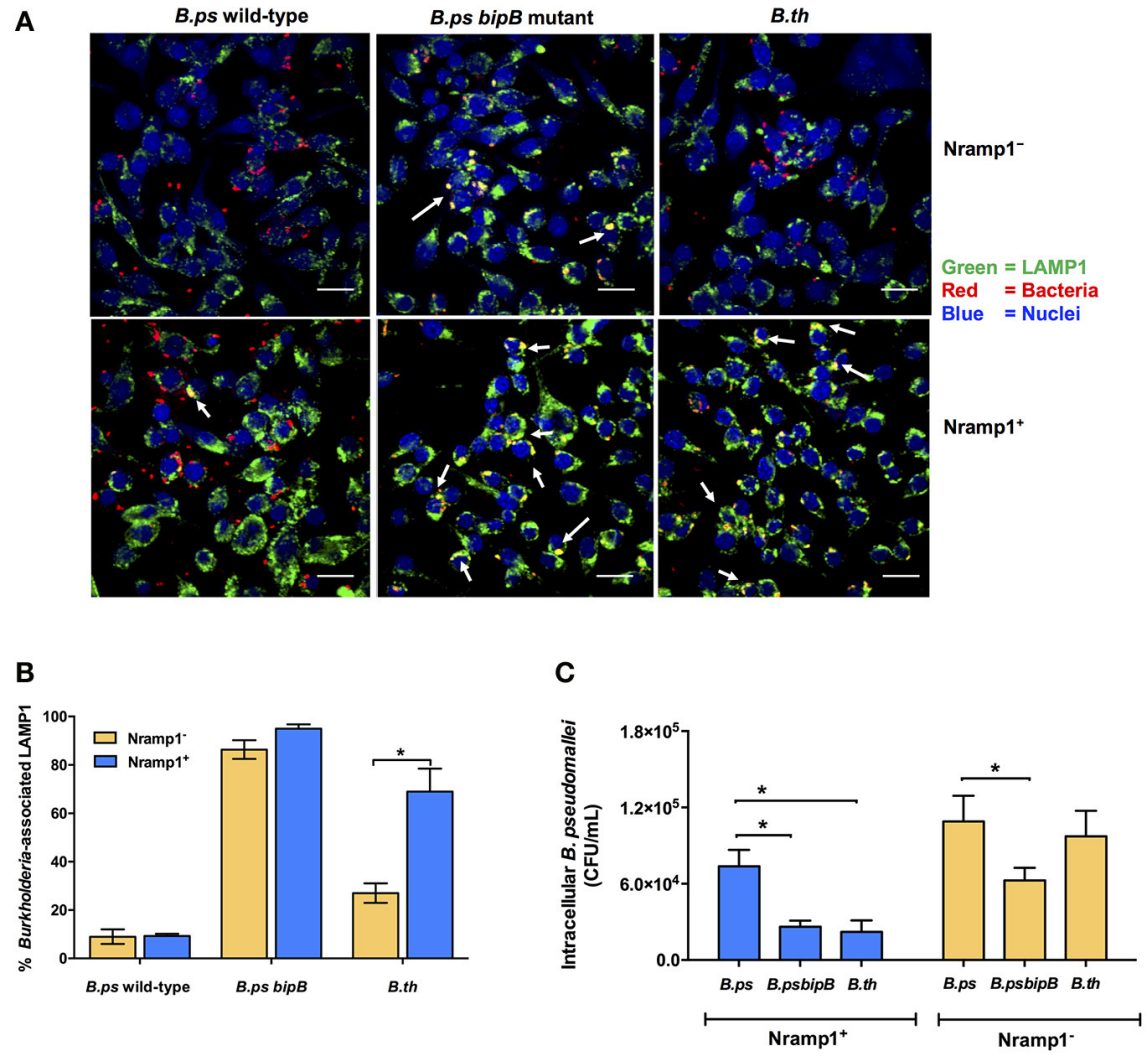
Analysis of *Burkholderia* co-localization with LAMP1 in macrophages expressing wild-type and mutated Nramp1. **(A)** Representative confocal micrographs of *Burkholderia*-infected Nramp1^+^ and Nramp1^−^ macrophages. **(B)** Quantitative analysis of *Burkholderia* co-localization with LAMP1 in Nramp1^+^ or Nramp1^−^ macrophages. *B. pseudomallei* wild-type, T3SS deficient *bipB* mutant and *B. thailandensis* were used to infect Nramp1^+^ or Nramp1^−^ cells. At 4 h p.i., the infected macrophage and bacterial cells were stained and analyzed by confocal microscopy. Host cell LAMP1 was stained green with rat monoclonal antibody (1D4B) and Alexa Fluor 488 goat anti-rat IgG antibody, and nuclei were stained blue with DAPI. Bacteria were stained red with mouse anti-*B. pseudomallei* monoclonal antibody (9D5) and Alexa Fluor 568 goat anti-mouse IgG antibody. The percentage of intracellular *Burkholderia* associated with LAMP1 was calculated by the number of bacteria associated with LAMP1/total number of intracellular bacteria × 100. **(C)** Comparative assessment of the net intracellular survival of a *B. pseudomallei bipB* mutant relative to wild-type strains within Nramp1^+^ and Nramp1^−^ macrophages at 6 h p.i. Results represent the mean ± SEM of three independent experiments. Asterisk indicates statistical significance (*P* ≤ 0.05, *t*-test) when comparing infected Nramp1^+^ cells with Nramp1^−^ cells.

One hypothesis to account for the difference in influence of Nramp1 on *B. pseudomallei* compared to *B. thailandensis* could relate to the ability of the bacteria to escape from the phagosome. We therefore used confocal microscopy to determine the level of escape from phagosomes by scoring co-localization of bacteria with LAMP1 (Figure 4A). At 4 h p.i. we found that c. 69% of intracellular *B. thailandensis* were associated with LAMP1^+^ vesicles in Nramp1^+^ cells (Figure 4B), this being a far greater proportion than the c. 9% of *B. pseudomallei* associated with LAMP1 in Nramp1^+^ cells at the equivalent time (Figure 4B). A *B. pseudomallei* mutant lacking the Bsa T3SS-3 translocon component BipB was included as control, as *bipB* mutant is known to exhibit delayed phagosome escape (Suparak et al., 2005). Of the intracellular *bipB* mutant bacteria analyzed, 95% were associated with LAMP1 (Figure 4B). These results suggested that *B. thailandensis* is less able to escape phagosome of Nramp1^+^ cells than *B. pseudomallei*. Significantly fewer *B. thailandensis* (27%; Figures 4A,B) were observed to be associated with LAMP1 in Nramp1^−^ cells, possibly as the bacteria are not controlled in this compartment in macrophages lacking functional Nramp1 and are therefore able to escape to the cytosol.

Consistent with the hypothesis that retention of *Burkholderia* in phagosomes leads to more effective killing of the bacteria, we observed that the *B. pseudomallei bipB* mutant was recovered from Nramp1^+^ macrophages at comparable levels to *B. thailandensis* (Figure 4C), The *bipB* mutant also exhibited moderate attenuation in Nramp1^−^ cells (Figure 4C), but the extent to which this reflects other cellular pathways for control of intracellular bacteria or other activities of T3SS-3 during intracellular life will be challenging to separate.

### MNGC formation in Nramp1^+^ and Nramp1^−^ macrophages

**Figure 5:**
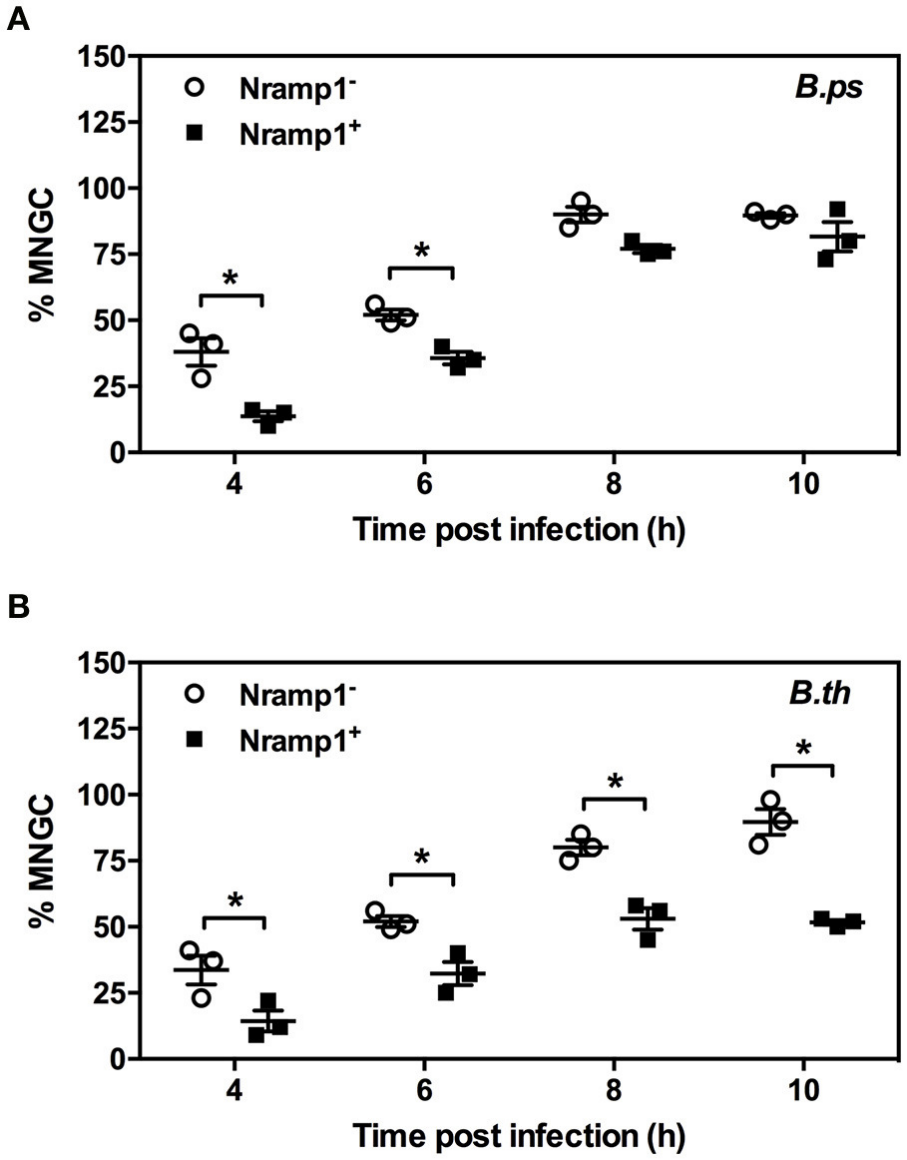
Impact of Nramp1 on *Burkholderia*-induced MNGC formation. The generation of MNGC in Nramp1^+/−^ macrophages after *B. pseudomallei* **(A)** or *B. thailandensis* **(B)** infection at different time points, as indicated. Results represent the mean ± SEM of three independent experiments. Asterisk indicates statistical significance (*P* ≤ 0.05, *t*-test) when comparing Nramp1^+^ cells with Nramp1^−^ cells.

MNGC formation was measured in activated (50 U/mL IFN-γ) *B. pseudomallei-* and *B. thailandensis*-infected Nramp1^+^ and Nramp1^−^ macrophages (Figure 5). At early stages of infection (4 and 6 h p.i.) we observed a significant difference in the percentage of nuclei in MNGC in Nramp1^+^ and Nramp1^−^ *B. pseudomallei* infected macrophages, with MNGC formation being higher in mutant macrophages (Figure 5A). This difference had waned at 8 and 10 h p.i., indicating an increase in MNGC formation in Nramp1^+^ macrophages that may be associated with increasing escape of *B. pseudomallei* from phagosomes over time. In contrast, the difference in MNGC formation between Nramp1^−^ and Nramp1^+^ macrophages was maintained throughout the 10 h infection period in *B. thailandensis*-infected macrophages (Figure 5B).

### Transcription of virulence-associated genes of *B. pseudomallei* is upregulated in Nramp1^+^ macrophages

Our study demonstrates that *B. pseudomallei* is better able to evade Nramp1-mediated control of bacterial replication than *B. thailandensis*. Among the bacterial factors that have been shown to be important virulence determinants of *B. pseudomallei* in murine models of melioidosis are Type VI secretion system cluster 1 (T6SS-1) (Burtnick et al., 2011), the Bsa Type III secretion system (T3SS-3) (Stevens et al., 2004) and BimA protein required for actin-based motility (Lazar Adler et al., 2015). Promoter-trap studies have indicated that Type VI secretion is induced during macrophage infection (Shalom et al., 2007). Negative regulation of *B. pseudomallei* T6SS-1 has been demonstrated in iron- and zinc-supplemented culture medium (Burtnick and Brett, 2013), but the role of these divalent cations in regulating expression upon interaction with host cells has not been studied.

**Figure 6:**
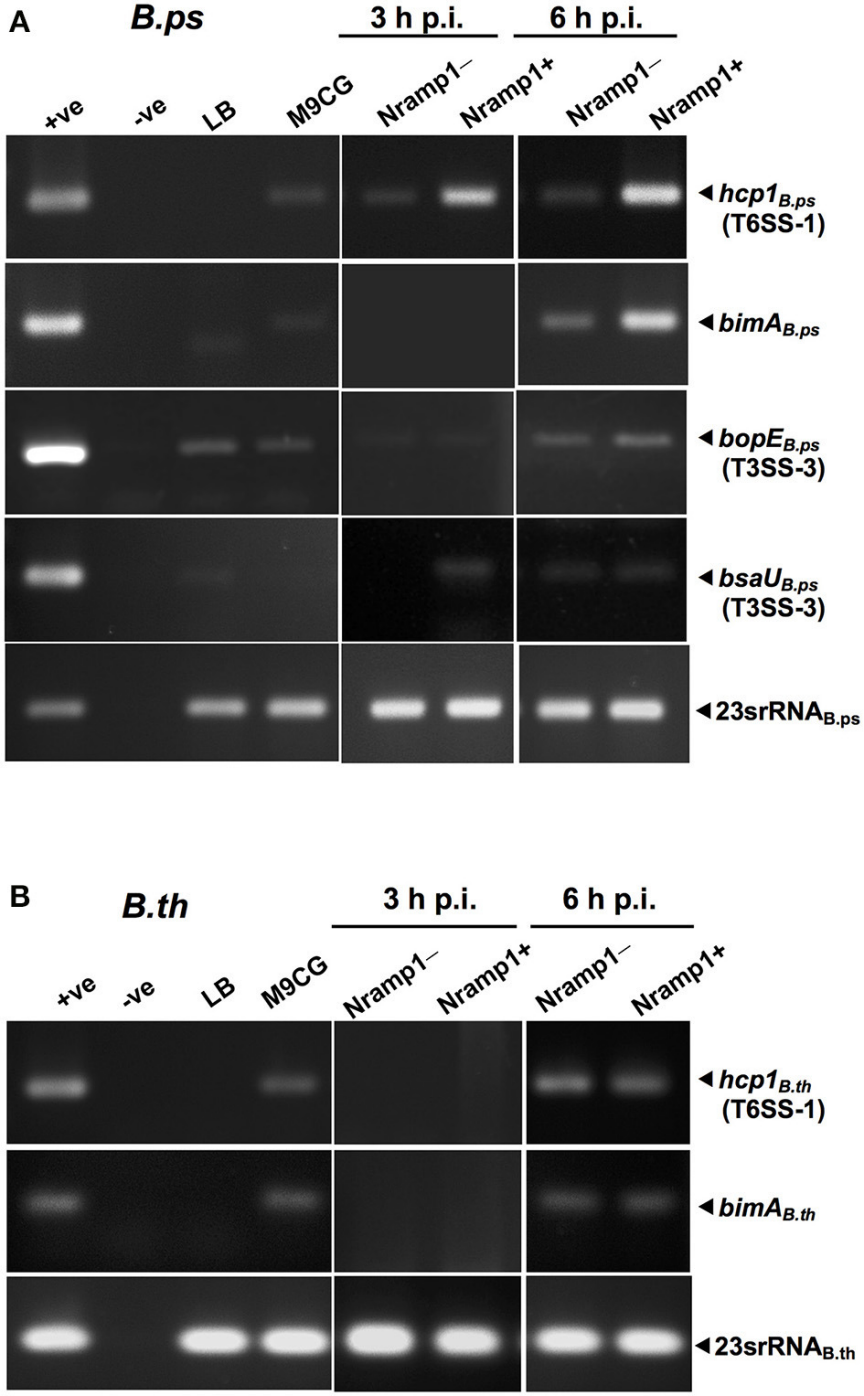
Effect of Nramp1 on transcription of *Burkholderia hcp1, bimA, bsaU* and *bopE* genes. The transcription of **(A)** *B. pseudomallei* or **(B)** *B. thailandensis* virulence genes was determined by RT-PCR in bacteria isolated from activated (50 U/mL IFN-γ) infected macrophages. RNA was harvested from bacteria isolated from infected macrophages at 3 or 6 h p.i., or from *Burkholderia* grown for 8 h in M9CG and LB medium. cDNA was subjected to PCR using specific primers and PCR products were visualized by gel electrophoresis. The image shown is representative of two independent experiments with similar results. All values have been normalized against the internal control, 23S rRNA gene. Positive controls were performed with genomic DNA. Negative controls were performed with RNA that had not been subjected to reverse transcription.

To determine the influence of functional wild-type Nramp1 on the induction of T6SS-1, transcription of *hcp1* was examined in activated (50 U/mL IFN-γ) *Burkholderia*-infected Nramp1^+^ and Nramp1^−^ cells. As shown in Figure 6A, the level of mRNA of *B. pseudomallei hcp1* in Nramp1^+^ cells was higher than in Nramp1^−^ cells at 3 and 6 h p.i., despite our observation that *B. pseudomallei* enters the cytosol at comparable levels in Nramp1^+^ and Nramp1^−^ cells as gauged by LAMP1 co-localization (Figures 4A,B). Similarly, transcription of *B. pseudomallei bimA*, located upstream of the T6SS-1 locus and co-regulated by the VirAG two-component sensory system, was induced in infected Nramp1^+^ cells when compared to Nramp1^−^ cells (Figure 6A). Bacteria cultured in rich LB broth or iron-limited minimal medium M9 supplemented with a carbon source and casamino acids (M9CG) were found to be negative and positive for transcription of these genes, respectively (Figure 6A). Transcription of the *B. pseudomallei bsaU* gene encoding a structural component of T3SS-3 appeared elevated at 3 h p.i in Nramp1^+^ cells compared to macrophages lacking functional Nramp1, though equivalent levels of the transcript were detected at 6 h p.i. (Figure 6A). Levels of the transcript for the T3SS-3 effectors *bopE* were slightly greater in Nramp1^+^ cells. However, quantitative analysis of amplified *bopE* gene band density indicated no significantly different between expression in Nramp1^+^ and Nramp1^−^ cells (Figure 6A). Transcription of the gene encoding 23S rRNA was observed under all condition tested, and showed no change over time. The absence of DNA contamination in RNA extracted samples was verified by PCR prior to cDNA synthesis (data not shown).

Unlike pathogenic *B. pseudomallei*, the expression of the *B. thailandensis hcp1* and *bimA* genes at 3 h p.i. was at the lower limit of detection, and showed no differences between Nramp1^+^ and Nramp1^−^ at 6 h p.i. (Figure 6B) This suggests that *B. thailandensis* did not respond differently to the environment within Nramp1^+^ cells as the pathogenic strain did, despite the presence of 90% nucleotide sequences identity in the coding and promoter regions between *B. pseudomallei* T6SS-1 (Accession no. BPSS1496-1511) and *B. thailandensis* (Accession no. BTH_II0855-0870). It has been reported in *B. pseudomallei* that *hcp1* and *bimA* genes were co-regulated (Burtnick et al., 2011). In Figure 6B, the expression levels of *hcp1* and *bimA* genes are similar suggesting that both genes may be co-regulated. This may imply the conservation of *hcp1* and *bimA* genes organization in *B. thailandensis* and *B. pseudomallei*, even though their precise location is unknown.

### Measurement of intracellular iron levels in Nramp1^+^ and Nramp1^−^ macrophages

**Figure 7:**
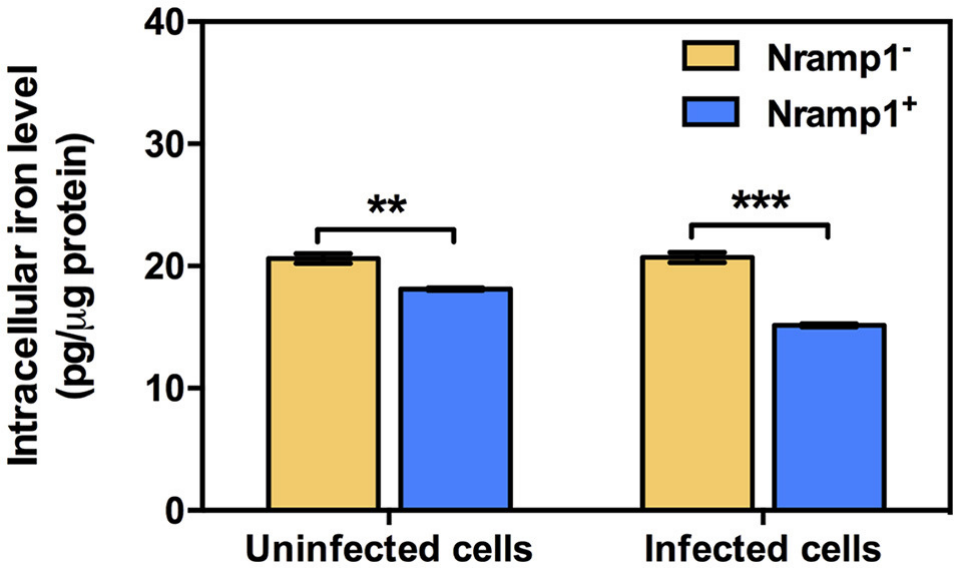
Measurement of intracellular iron levels in Nramp1^+^ and Nramp1^−^ macrophages. The total cellular iron level of *Burkholderia*-infected cells was measured by means of atomic absorption spectrometry and normalized for protein content. Results represent the mean ± SEM of three independent experiments. Asterisk indicates statistical significance (*P* ≤ 0.05, *t*-test) when comparing between Nramp1^+^ and Nramp1^−^ cells.

Previous studies have shown that once *B. pseudomallei* escapes from the phagosome and enters the cytosol, highly reducing conditions induce expression of T6SS-1 *via* VirAG regulation activated by host glutathione (GSH) (Wong et al., 2015). We hypothesized that upregulation of *Burkholderia* T6SS-1 may be a consequence of differing levels of intracellular iron within Nramp1^+^ compared to Nramp1^−^ cells. Total intracellular iron content of Nramp1^+^ and Nramp1^−^ cells in response to *Burkholderia* infection was quantified by atomic absorption spectrometry and normalized to total protein concentration. Activated (50 U/mL IFN-γ) *B. pseudomallei-*infected Nramp1^+^ cells showed a significant reduction of the total cellular iron level when compared to Nramp1^−^ cells (Figure 7). Since iron limitation is a known regulator of *hcp1* (Burtnick and Brett, 2013), it is plausible that the reduced iron concentration detected in Nramp1^+^ macrophages may be a potential explanation for activation of the VirAG-coregulated *hcp1* and *bimA* genes.

## Discussion

**Figure 8:**
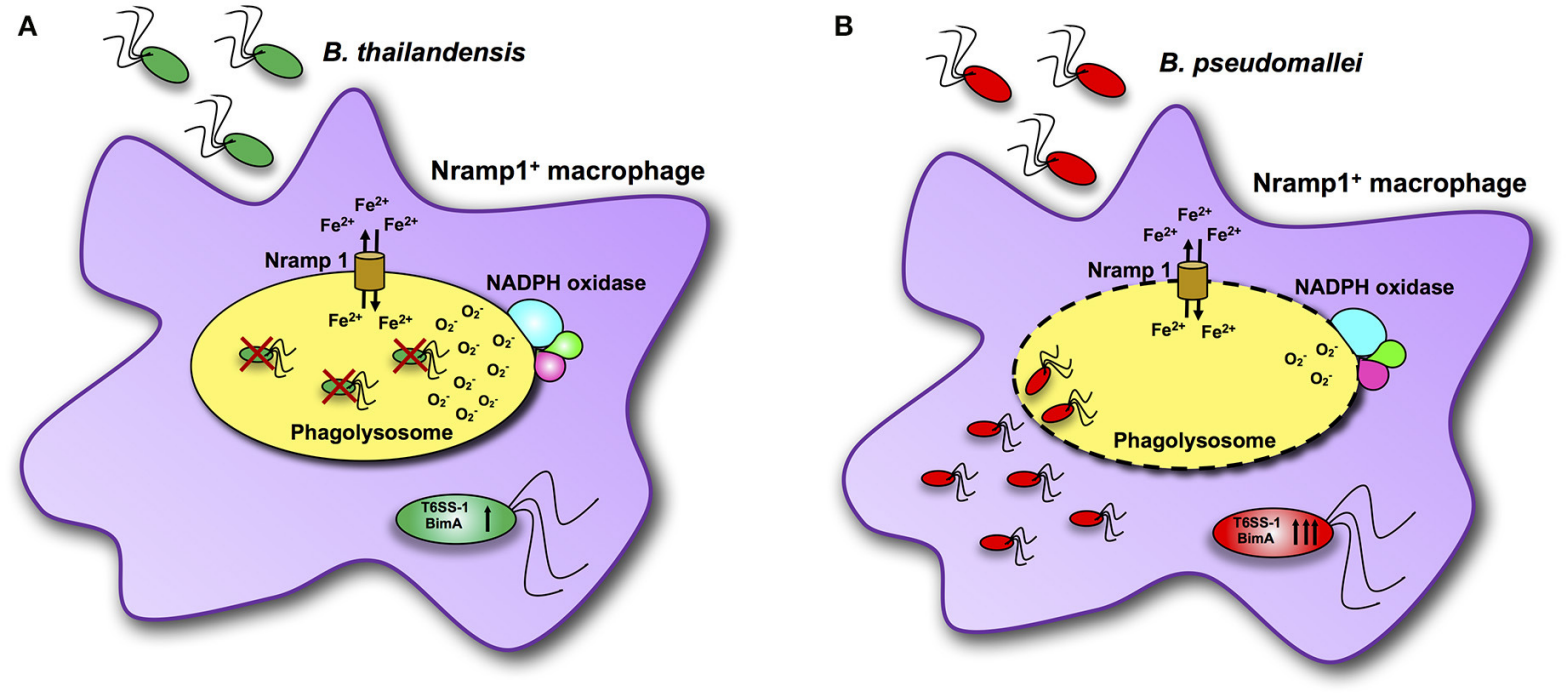
Scheme of interactions between *Burkholderia* bacteria and Nramp1 activity in macrophages. **(A)** Nramp1 restricts growth of non-pathogenic *B. thailandensis* within the phagoslysome *via* an NADPH oxidase-mediated mechanism. **(B)** Pathogenic *B. pseudomallei* are able to evade Nramp1- and NAPDH oxidase-mediated killing in macrophages by escaping more rapidly from the phagolysosome. Elevated levels of transcription of virulence-associated genes occur in *B. pseudomallei*-infected Nramp1^+^ cells, possibly as a consequence of escaping to the cytosol to a greater extent that *B. thailandensis* and encountering a more iron-restricted environment.

Previous studies have demonstrated that control of *B. pseudomallei* infection involves innate immune responses, a key component of which is activation of macrophages and neutrophils to kill bacteria (Lazar Adler et al., 2009). The Nramp1 is known to have many pleiotropic effects on macrophage activation, leading to efficient bacterial clearance for a number of intracellular bacterial and protozoan pathogens (Wessling-Resnick, 2015). Here we present data on the impact of some of these Nramp1-regulated pleiotropic effects on clearance of *Burkholderia* spp., in particular in relation to the differences in virulence of *B. pseudomallei* compared to *B. thailandensis* as summarized in Figure 8. Nramp1 controls growth of *B. thailandensis* within the phagoslysome *via* an NADPH oxidase-mediated mechanism (Figure 8A). In contrast, *B. pseudomallei* are able to evade Nramp1- and NAPDH oxidase-mediated killing in macrophages by escaping more rapidly from the phagolysosome (Figure 8B). Increased levels of transcription of virulence-associated genes occur in *B. pseudomallei*-infected Nramp1^+^ cells, possibly as a consequence of escaping to the cytosol to a greater extent that *B. thailandensis* and encountering a more iron-restricted environment.

First we show that functional wild-type Nramp1 can control the intracellular replication of *B. thailandensis*, but not *B. pseudomallei*, within IFN-γ stimulated macrophages. The action of Nramp1 in determining the response to macrophage activation by IFN-γ is well established (Atkinson et al., 1997; Barrera et al., 1997; Fritsche et al., 2003; Monack et al., 2004). Furthermore, the growth restriction of *B. thailandensis* in macrophages expressing wild-type Nramp1 was dependent upon the presence of functional NADPH oxidase but not inducible nitric oxide synthase. It is acknowledged that whilst DPI inhibits NADPH oxidase-mediated ROS production it may also affect other flavor enzymes, such as xanthine oxidase and nitric oxide synthase (NOS) (O'Donnell et al., 1993). We consider it unlikely that indirect effects on NOS explain the impact of DPI on control of *B. thailandensis* in Nramp1^+^ macrophages as the iNOS inhibitor aminogaunine had no significant effect. The absence of a role for NOS in control if intracellular *Burkholderia* is consistent with earlier studies showing that *Nos2*^−/−^ gene disruption did not affect Nramp1-mediated early resistance to *Leishmania donovani* or *S*. Typhimurium infection in murine models of these diseases (White et al., 2005). In the case of *S*. Typhimurium, further studies using Nramp1 transfected cells suggest that Nramp1-mediated alterations in ROS production stimulate NF-kB activity and transcription of Lcn2, a peptide that exerts antimicrobial activity by scavenging iron-loaded bacterial siderophores and mediating iron efflux from macrophages (Fritsche et al., 2012). For mycobacteria, it was earlier proposed (Zwilling et al., 1999) that Nramp1-mediated transport of ferrous ions (Fe^2+^) from the cytosol into the bacteria containing phagosome leads to production of more toxic ROS radicals *via* the Fenton and Haber-Weiss mechanism (Zwilling et al., 1999).

In our study we observed enhanced production of TNF-α and IL-6 in *B. thailandensis* (but not *B. pseudomallei*) infected macrophages expressing wild-type Nramp1 compared to mutant Nramp1. These are cytokines which are considered to be involved in mitochondrial ROS generation *via* RIP1–RIP3-dependent pathways (Roca and Ramakrishnan, 2013). The NADPH oxidase inhibitor DPI used in our study has also been shown to inhibit the production of mitochondrial ROS (Li and Trush, 1998), while recent studies have drawn attention to the role of mitochondrial ROS as another important antimicrobial mechanism against mycobacteria (Roca and Ramakrishnan, 2013). Further research will be required to determine which of these mechanisms are contributing to the role of Nramp1 in determining growth restriction of *B. thailandensis in vitro* or *in vivo*, or whether other pleiotropic mechanisms associated with Nramp1 activity are at play.

Next, to understand the ability of *B. pseudomallei*, but not the closely related *B. thailandensis*, to avoid Nramp1-mediated antimicrobial activity we focused our attention on the dynamics of bacterial escape from phagosomes. Co-localization of *B. pseudomallei* with the phagosome marker LAMP1 was far lower than for *B. thailandensis* at the time intervals sampled, indicating that it more efficiently escapes from a cellular compartment where Nramp1-mediated oxidative killing occurs. Phagosome escape is known to be influenced by the Bsa Type III secretion system (Stevens et al., 2002) and it is plausible that differences in the dynamics of phagosome escape between *B. pseudomallei* and *B. thailandensis* reflect the fact that the *bsa* locus is repressed in *B. thailandensis* (Moore et al., 2004), and/or that Bsa T3SS components predicted to interact with the host cell differ in sequence (Kim et al., 2005). A *B. pseudomallei bipB* mutant was confined to phagosomes and killed to a similar extent to *B. thailandensis* in Nramp1^+^ cells, but also exhibited attenuation in Nramp1^−^ macrophages possibly owing to other killing mechanisms or the need for the Bsa T3SS in other aspects of intracellular life. Our finding that Nramp1 has distinct effects on intracellular survival and gene expression in *B. thailandensis* vs. *B. pseudomallei* is important, as it has been proposed that the former species can be used as a surrogate for the pathogenic strain which requires biosafety level 3 containment (Haraga et al., 2008).

Once in the cytoplasm, *B. pseudomallei* is exposed to a different intracellular niche, where distinct environmental cues exist that regulate gene expression. Expression of the T6SS-1 effector Hcp1 and the BimA protein mediating actin-based motility, is required for cell-to-cell spreading and MNGC formation, a hallmark of *B. pseudomallei* infection (Kespichayawattana et al., 2000; Stevens et al., 2005). Earlier studies have shown that *B. pseudomallei* T6SS-1 gene expression is negatively regulated under iron rich conditions during *in vitro* growth (Burtnick and Brett, 2013). Nramp1^+^ cells become iron limited due to a significant reduction in transferrin receptor 1 (TfR1)-mediated iron uptake and ferroportin 1 (Fpn1)-mediated iron export (Fritsche et al., 2007). In contrast, Nramp1^−^ cells remain iron sufficient. Our data confirm a reduction in intracellular iron in Nramp1^+^ compared to Nramp1^−^ macrophages, which may be associated with the enhanced induction of the T6SS-1-encoded *hcp1* gene in *B. pseudomallei-*infected Nramp1^+^ macrophages. Induction of *bimA* (which is co-regulated with T6SS-1 by VirAG) was also observed in Nramp1^+^ macrophages infected with *B. pseudomallei* at 6 h p.i, when escape into the cytosol is known to have occurred (Stevens et al., 2002). It is important to note that induction of *hcp1* and *bimA* occurred to a greater extent in Nramp1^+^ macrophages, even though the extent of co-localization of *B. pseudomallei* with LAMP1 was comparable in macrophages expressing wild-type and mutant Nramp1. This implies that in both cell types the bacteria escape to the cytosol at an equivalent level, and that the differences in gene expression may therefore reflect Nramp1-dependent differences in the environmental cues they encounter.

It is plausible that the reduced levels of intracellular iron detected in Nramp1^+^ cells are responsible for the observed induction of *hcp1* and *bimA* expression, however further studies will be required to determine the cytosolic concentration of iron in cells expressing wild-type or mutated Nramp1 and test whether the differences observed are sufficient to trigger a change in *hcp1* and *bimA* expression *in vitro*. Moreover, studies with *hcp1* or *bimA* mutants in Nramp1^+^ or Nramp1^−^ macrophages may help to elucidate whether the observed differences in MNGC formation occur through Type VI secretion- and/or actin-based motility-dependent pathways. However, it should be noted that at many time intervals cell fusion occurred to a greater extent in macrophages lacking functional Nramp1 and at no time interval tested was MNGC formation greater in Nramp1^+^ cells. Nevertheless, our study indicates for the first time that Nramp1- and NAPDH oxidase-mediated control of intracellular *Burkholderia* differs between non-pathogenic and pathogenic species, and indicates that Nramp1 can affect the expression of virulence-associated genes in a manner that appears independent of escape from phagosomes.

## Author contributions

VM, JB, and SK designed the research. VM performed experiments and analyzed data. VM, PW, and VS performed infection experiments with *B. pseudomallei*. NC developed monoclonal antibody to *B. pseudomallei* and *B. thailandensis* for immunofluorescence detection. MS critically revised the manuscript. VM, JB, and SK wrote the manuscript. JB and SK conceptualized the study. All authors read and approved the final version of the manuscript.

### Conflict of interest statement

The authors declare that the research was conducted in the absence of any commercial or financial relationships that could be construed as a potential conflict of interest.
